# Provision of Oral Health Care to Children under Seven Covered by *Bolsa Família* Program. Is This a Reality?

**DOI:** 10.1371/journal.pone.0161244

**Published:** 2016-08-18

**Authors:** Krishna Andréia Feitosa Petrola, Ítalo Barroso Bezerra, Érico Alexandro Vasconcelos de Menezes, Paola Calvasina, Maria Vieira de Lima Saintrain, Anya Pimentel G. F. Vieira-Meyer

**Affiliations:** 1 Municipal Department of Health, Fortaleza, Ceará, Brazil; 2 University of Fortaleza (UNIFOR), Ceará, Brazil; 3 Oswaldo Cruz Foundation (FIOCRUZ), Ceará, Brazil; 4 Public Health Masters Program, UNIFOR, Ceará, Brazil; 5 Oswaldo Cruz Foundation (FIOCRUZ), and Family Health Masters Program, FIOCRUZ, Brazil; University of Washington, UNITED STATES

## Abstract

Over the last decade, there has been a great improvement in the oral health of Brazilians. However, such a trend was not observed among five-year-old children. Dental caries are determined by the interplay between biological and behavioral factors that are shaped by broader socioeconomic determinants. It is well established that dental disease is concentrated in socially disadvantaged populations. To reduce social and health inequalities, the Brazilian government created Family Health Program (ESF), and the *Bolsa Família* Program, the Brazilian conditional cash transfer program (*Bolsa Família* Program). The aim of this study was to examine the oral health care and promotion provided by the Family Health Teams to children and caregivers covered by the *Bolsa Família* Program. Data was collected through interviews with three groups of participants: 1) dentists working for the Family Health Program; 2) Family Health Program professionals supervising the *Bolsa Família* Program health conditionalities (*Bolsa Família* Program supervisors); and 3) parents/caregivers of children covered by the *Bolsa Família* Program. A pretested questionnaire included sociodemographic, *Bolsa Família* Program, oral health promotion, dental prevention and dental treatment questions. The results showed that most dentists performed no systematic efforts to promote oral health care to children covered by the *Bolsa Família* Program (93.3%; n = 69) or to their parents/caregivers (74.3%; n = 55). Many dentists (33.8%) did not provide oral health care to children covered by the *Bolsa Família* Program because they felt it was beyond their responsibilities. Nearly all *Bolsa Família* Program supervisors (97.3%; n = 72) supported the inclusion of oral health care in the health conditionality of the *Bolsa Família* Program, but 82.4% (n = 61) stated they did not promote oral health activities to children covered by the Bolsa Família Program. Children in the routine care setting were more often referred to dentists than children covered by the Bolsa Familia Program (*p*≤0.001). Parents/caregivers (99.2%; n = 381) agreed that oral health care is important and 99.5% (n = 382) would like their children to be seen regularly. **Conclusions:** No collaboration was observed between the *Bolsa Família* Program and the Family Health Program with regard to the provision of oral health care. Making oral health care a *Bolsa Família* Program conditionality may reduce oral health care inequalities for extreme poor children under seven in Brazil.

## Introduction

The field of dentistry has over the past century witnessed numerous advances in research and biomedical technology, leading to improvements in health and overall well-being worldwide [1]. However, despite these achievements, dental caries―affecting 60–90% of school children around the world―remains a serious challenge for public health authorities [2].

The prevalence of dental caries is strongly shaped by the social determinants of health. In general, socioeconomically disadvantaged communities present higher levels of dental caries, [2] in part associated with limited access to dental services [3]. Although the human development index of Brazil (0.74) has improved considerably over the past two decades, extreme poverty still afflicts 8.5% of the population, more than half of which (59%) is in the Northeast [4]. Over the same time period, dental caries have significantly decreased among Brazilians. However, such a decrease was not observed among five-year-old children. Data from the most recent Brazilian Oral Health Survey (2010) [5] revealed a decrease of only 18%, in the prevalence of dental caries among five-year-old children over the period of seven years. In addition, 80% of decayed deciduous teeth remained untreated in this population [5].

The burden of dental disease is highly concentrated in socially disadvantaged populations, especially in the Northeast of Brazil, one of the poorest regions of the country [6,7]. Dental caries is a preventable disease, which can be controlled with a comprehensive approach including home and professional care [8]. Because infants and toddlers are not able to care for their own oral health, parents and caregivers play a pivotal role in supporting early oral health—by establishing positive oral health care family routines, implementing proper nutrition, ensuring appropriate amounts of fluoride, and taking children to the dentist by age one. Therefore, much can be done for children’s oral health when patients, parents, health care providers and policy makers work towards an environment of health promotion and prevention [9].

In order to reduce social and health inequalities, the Brazilian government created the Family Health Program (*Estratégia Saúde da Família*—ESF), and the *Bolsa Família* Program (PBF), the Brazilian Conditional Cash Transfer (CCT) Program. The Family Health Program is the primary health care strategy implemented by the Brazilian universal health care system, [10]. This program provides a broad range of primary health care services delivered by a multidisciplinary team comprised of a physician, a nurse, a nursing assistant, community health workers and an Oral Health Team—dentist and dental assistant with or without dental hygienists [11]. Each team is assigned to a geographical area and is then responsible for the health of the population living in this area, providing primary health care services [12], and making referrals to other levels of care as required. The health professional teams must work under the aegis of primary health care principles; providing basic health care, promoting health activities and preventing diseases. Regarding oral health, the Family Health Program/Oral Health Teams are responsible for providing comprehensive oral health care (i.e., oral health promotion, preventive dental care and dental rehabilitation treatment) for patients of all ages. In 2011, Family Health teams covered 94% of municipalities in Brazil, corresponding to 53% of the population [13]. In 2015, there were over 21,000 Oral Health Team working in the Family Health Program providing care at Public Primary Health Care (PPHC) Facilities around the country [14].

The *Bolsa Família* Program is a Conditional Cash Transfer Program (CCT) intended to help emancipate socioeconomically vulnerable families [15] by breaking the cycle of poverty and extreme poverty. Financial benefits are conditional upon compliance with certain actions related to education, health care, nutrition and social service. Actions in health care for children under seven years of age are centered on adherence to vaccination schedules and regular health check-ups at the Public Primary Health Care facilities [16]. Several studies worldwide have demonstrated that conditional cash transfer programs positively impact children's health through two pathways: 1) through the increase in the use of preventive services, immunization coverage, and promotion of healthy behavior change [17–20] and, 2) through the effect of the increased income in extremely poor families [13,21].

Oral health is not included as a health conditionality in the Brazilian Conditional Cash Transfer Program, however, children under seven years of age are expected to visit the Public Primary Health Care facility every six months to comply with the *Bolsa Família* health conditionality. The Oral health teams work in the same Public Primary Health Care facilities where children covered by the *Bolsa Família* Program perform their check-ups. Thus, the Oral Health Teams have the opportunity to provide oral health care for the Children covered by the *Bolsa Família* Program, who, due to low socioeconomic position, are at higher risk of developing dental diseases [2]. Still, it is unknown, the path of oral health care available for Children covered by the *Bolsa Família* Program

The purpose of this study was to investigate the provision of oral health care to children under seven years old covered by the Bolsa Família Program by the Family Health Program in Fortaleza, Northeast of Brazil from the perspective of caregivers and health professionals. Fortaleza is the fifth most populous city in the country [22] and is among the top 20 most socially inequitable cities in the world [23]. In addition, the city has the largest absolute number of children benefited by the *Bolsa Família* Program among the nine state capitals of Northeastern Brazil [24].

## Methods

The study was conducted at 74 Public Primary Health Care facilities in Fortaleza served by Family Health Program/Oral Health Team. Information was collected through interviews with three groups of participants: 1) dentists working on Family Health Program/Oral Health Team; 2) Family Health Program professionals supervising compliance with *Bolsa Família* Program health conditionalities (in this study referred to as *Bolsa Família* Program supervisors); and 3) parents/caregivers of children under seven covered by the *Bolsa Família* Program.

The inclusion criterion for the first two groups of participants was to have been employed by the Family Health Program in Fortaleza for at least twelve consecutive months. The inclusion criterion for the third group of participants was to have been an effective caregiver of a child under seven covered by the *Bolsa Família* Program in Fortaleza for at least six consecutive months.

The Public Primary Health Care facility was the unit of analysis in the first two groups. Sample size was determined by random sample size calculation, considering a sample error of 5%, a 95% confidence interval and the total number of Public Primary Health Care facilities in Fortaleza (N = 91). Thus, the final sample consisted of 74 facilities. The facilities to be visited were randomly selected through a raffle. Each health facility has one *Bolsa Família* Program Supervisor, who was interviewed. When the health facility had more than one dentist, another dentist was selected at random. In some cases, a second trip to the health facility was necessary, as the professional accepted to participate in the research, but did not have the time to answer the questionnaire during the first visit.

The sample of parents/caregivers (n = 384) was estimated based on the total number of children under seven years old covered by the *Bolsa Família* Program in Fortaleza (n = 130,000) (2012 data provided by the Municipal Department of Social Assistance), considering a sample error of 5% and a 95% confidence interval. Thus, a sample of 384 parents/caregivers was estimated. As children covered by the *Bolsa Família* need to attend the health facility every six months for the regular health check-ups (i.e., get weighed and measured for height), this was the moment chosen to contact their parents/caregivers. Therefore, during the health facility visit, parents/caregivers accompanying children to their routine *Bolsa Família* Program health visit were asked to participate in the research. Knowing that 74 Public Primary Health Care facilities were to be visited during the research data collection, approximately five parents/caregivers per facility (384/74) were invited to participate in the study. If the data collection was not concluded on the first visit, the health unit was revisited in the following business days until completion of sample was achieved.

Over a period of 6 months, the three groups were interviewed each with a different questionnaire containing questions about socioeconomic status, demographics, the *Bolsa Família* Program and oral health promotion, prevention and treatment. The questionnaires were created by the researchers based on the study objectives. This questionnaire was previously pilot tested in Public Primary Health Care facilities not included in the study to adjust for clarity content, intelligibility, length, and overall adequacy in assessing the research objectives. Three researchers (KAFP, IBB, EZVM) were trained by the main investigator (APGFVM) to collect the data. The response rate was high—100% for the *Bolsa Família* Program supervisors, and above 90% for the dentist and child/caregiver groups. Only one dentist refused to participate in the research, and gave no reason for it. In this specific case, a second dentist from the same health facility was chosen at random to be interviewed. The few caregivers that refused to participate in the research alleged lack of time as the main reason.

Data were analyzed using the software SPSS 20.0 (SPSS Inc., Chicago, IL, USA). The study protocol was previously approved by the research ethics committee of the Ceará State University (UECE). All participants gave their informed written consent. Univariate descriptive statistics on sociodemographics, provision of oral health care, knowledge of the *Bolsa Família* Program, and the agreement over the establishment of an oral health conditionality in the *Bolsa Família* Program were performed for each study group participants. Bivariate statistics using the Pearson Chi-squared test [interpreted at 5% significance level] were performed on: 1) the willingness to include oral health conditionality in the *Bolsa Família* Program by sociodemographics; 2) the development of oral health promotion activities by sociodemographics. Additionally, we assessed if there were differences regarding provision of oral health care among dentists who only work at Family Health Program and those who work a third shift elsewhere (both with the same work shift at the Family Health Program).

## Results

A total of 532 interviewees participated in the study (74 dentists, 74 *Bolsa Família* Program supervisors and 384 parents/caregivers). The dentists were aged 38.1±7.3 years on average (range:30–61); most were female (81.1%;n = 60) and most were specialists (87.8%;n = 65), with Family Health as the most frequently reported specialty (27.2%;n = 20). Many (41.9%;n = 31) had worked for the Family Health Program for over 10 years. Over half the dentists (51.4%;n = 38) did not work exclusively for the Family Health Program (Table 1).

**Table 1:** Sociodemographic characteristics of the study sample. Fortaleza, 2014.

	Dentists		PBF supervisors		Parents/caregivers	
	n	%	n	%	n	%
**Sample**	74	100	74	100	384	100
**Gender**						
Female	60	81.1	66	89.2	376	97.9
Male	14	18.9	8	10.8	8	2.1
**Marital status**						
Married	57	77.0	48	64.9	116	30.2
Single	10	13.5	16	21.6	133	34.6
Divorced	6	8.1	5	6.8	14	3.6
Widow(er)	1	1.4	2	2.7	6	1.6
Others	0	0	3	4.1	115	29.9
**Gross monthly household income**						
Up to 1 MW (BRL 678)	-	-	-	-	270	70.3
1–2 MWs (BRL 678–1356)	-	-	-	-	111	28.9
3–4 MWs (BRL 1356–2034)	-	-	-	-	3	.8
5–9 MWs (BRL 2034–6102)	24	32.4	29	39.2	-	-
9–12 MWs (BRL 6102–8136)	32	43.2	31	41.9	-	-
Over 12 MWs (>BRL 8136)	18	24.3	14	18.9	-	-
**Level of schooling**						
No formal schooling	-	-	-	-	13	3.4
Elementary school dropout	-	-	-	-	169	44.0
Elementary school completed					48	12.5
High school dropout	-	-	-	-	52	13.5
High school completed	-	-	-	-	97	25.3
College degree	74	100-	74	100-	5	1.3
Specialization/post-graduation	74	100	70	94.6		
**Most cited fields of specialization**						
Family health	20	27.2	39	52.8	-	-
Public health	5	6.8	8	10.8	-	-
Orthodontics	15	20.2	-	-	-	-
Cosmetic dentistry	7	9.4	-	-	-	-
Occupational nursing	-	-	4	5.4	-	-
**Time working for the ESF**						
1 ├ 2 years	5	6.8	1	1.4	-	-
2 ├ 5 years	10	13.5	12	16.2	-	-
5 ├ 10 years	28	37.8	11	14.9	-	-
10 ├ 15 years	26	35.1	34	45.9	-	-
≥ 15 years	5	6.8	12	16.2	-	-
Does not work exclusively for the ESF	38	51.4	29	39.2	-	-

ESF = Estratégia Saúde da Família (Family Health Program)

MW = Minimum wage

BRL = Brazilian currency (Real)

Approximately 83% (n = 61) of the dentists considered the PBF important for childrenʼs health. Most were aware of (73%;n = 54) and agreed with (66.3%;n = 49) the PBF conditionalities (Table 2). The vast majority (90%;n = 66) agreed with the inclusion of oral health care in the conditionality of the PBF in the form of semiannual check-ups (50%;n = 37) (Table 2). However, most dentists performed no systematic efforts to provide oral health promotion or prevention care (fluoride therapy and/or instruction in oral hygiene) to children covered by the *Bolsa Família* Program (93.2%;n = 69) or their parents/caregivers (74.3%;n = 55) (Table 3). Many dentists (33.8%) did not specifically provide oral health care (oral health promotion, preventive and rehabilitating care) to children covered by the *Bolsa Família* Program because they felt it was beyond their responsibilities as Family Health Program professionals and due to the lack of integration between the two programs. In addition, according to 81.1% (n = 60), the infrastructure, human resources and dental supplies required for such actions were not available in the workplace (Table 3).

**Table 2:** Questions regarding *Bolsa Família* Program (PBF). Fortaleza, 2014.

	Dentists		PBF supervisors		Parents/caregivers	
	n	%	n	%	n	%
**Sample**	74	100	74	100	384	100
**Are you aware of the PBF conditionalities?**						
Yes	21	28.4	-	-	362	94.3
No	20	27.0	-	-	22	5.7
To some extent	33	44.6	-	-	-	-
**Do you agree with the PBF conditionalities?**						
Yes	39	52.7	46	62.2	344	89.6
No	5	6.8	6	8.1	9	2.3
To some extent	10	13.5	22	29.7	9	2.3
**If yes, for what reasons?**						
It is important for childrenʼs health and development	-	-	-	-	171	44.5
Itʼs a form of control for the PBF	-	-	-	-	40	10.4
It increases parentsʼ commitment to childrenʼs health	-	-	-	-	113	29.4
It encourages health care, organizes and facilitates access	-	-	-	-	7	1.8
Health and education are rights of children and parents	-	-	-	-	4	1.0
Other	-	-	-	-	9	2.3
**Is the PBF important for the health of children <7 years?**						
Yes	46	62.2	46	62.2	375	97.7
No	6	8.1	4	5.4	1	0.3
To some extent	15	20.3	24	32.4	8	2.0
Doesnʼt know	7	9.5	0	0	0	0
**Would you like your children to see the dentist regularly as part of the PBF conditionality?**						
Yes	-	-	-	-	382	99.5
Indifferent	-	-	-	-	2	0.5
**Are oral health check-ups important for children covered by the PBF?**						
Yes	68	91.9	72	97.3	381	99.2
No	4	5.4	0	0	2	0.5
To some extent	2	2.7	2	2.7	1	0.3
**Should oral health care be made a PBF conditionality?**						
Yes	66	89.2	72	97.3	341	88.8
No	8	10.8	2	2.7	36	9.4
To some extent	0	0	0	0	0	0
Doesnʼt know	0	0	0	0	7	1.8
**If so, why?**						
It encourages parents to care for childrenʼs oral health	23	31.1	24	32.4	65	16.9
It is part of overall health	21	28.4	16	21.6	56	14.6
It facilitates access to children	6	8.1	4	5.4	107	27.9
Preventive care should start as early as possible	6	8.1	16	21.6	25	6.5
It helps prevent problems and favors health and development	9	12.2	10	13.5	69	18.0
Other	1	1.4	2	2.7	19	4.9
**Suggested oral health-related conditionalities**						
Visit the dentist biannually	37	50.0	47	63.5	-	-
Visit the dentist annually	10	13.5	9	12.2	-	-
Other	5	6.8	16	21.7	-	-
**How many of your children attend the facility as a PBF conditionality?**						
One	-	-	-	-	277	72.1
Two	-	-	-	-	85	22.1
Three	-	-	-	-	14	3.6
Four	-	-	-	-	6	1.6
Five	-	-	-	-	2	0.5
**Kinship with child**						
Mother	-	-	-	-	327	85.1
Father	-	-	-	-	7	1.8
Grandmother	-	-	-	-	47	12.2
Other	-	-	-	-	3	0.9

**Table 3:** Questions regarding oral health care provided by the Family Health Program. Fortaleza, 2014.

	Dentists		PBF Supervisors	
	N	%	N	%
**Sample**	74	100	74	100
**Are the structure, resources and supplies required for oral health promotion, prevention and rehabilitation available in the work place?**				
Yes	14	18.9	24	32.4
No	34	45.9	36	48.6
To some extent	26	35.1	14	18.9
**Do you participate in oral health promotion and prevention for children <7 years?**				
In schools/kindergartens?				
Always	32	43.2	-	-
Sometimes	37	50.0	-	-
Never	5	6.8	-	-
Outside schools/kindergartens?				
Always / Yes	19	25.7	50	67.6
Sometimes	45	60.8	-	-
Never / No	10	13.5	24	32.4
**Specifically for children <7 years covered by the PBF?**				
Yes	5	6.8	13	17.6
No	69	93.2	61	82.4
**If so, what actions are performed?**				
Health promotion	5	6.8	-	-
Supervised brushing	3	4.1	-	-
Fluoride therapy	2	2.7	-	-
Educational activities	3	4.1	12	16.2
Other	2	2.8	-	-
**If not, why?**				
Never thought about it	22	29.7	18	24.3
Lack of time	5	6.8	18	24.3
Lack of collaboration between programs	25	33.8	4	5.4
The PBF does not demand it	10	13.5	17	23.0
Other	29	39.5	23	31
**Do you participate in oral health promotion and prevention for the parents/caregivers of children <7 years?**				
In schools/kindergartens?				
Always	4	5.4	-	-
Sometimes	40	54.1	-	-
Never	30	40.5	-	-
Outside schools/kindergartens?				
Always / Yes	28	37.8	38	51.4
Sometimes	36	48.6	-	-
Never / No	10	13.5	36	48.6
Do you participate in oral health promotion and prevention for the parents/caregivers of children <7 years covered by the PBF?				
Always	6	8.1	18	24.3
Sometimes	13	17.6	-	-
Never	55	74.3	56	75.7
If not, why?				
Never thought about it	18	24.3	26	35.1
Lack of collaboration between programs	30	40.5	4	5.6
The PBF does not demand it	6	8.1	15	20.3
Lack of time	3	4.1	15	20.3
Other (e.g., PBF group not identified, Demand is too high, **Only perform activities for children, Parents only go to the health facilities to have their children measured—height and weight).**	29	39.2	14	18.9
**How often do parents/caregivers bring their children <7 years to the facility as part of the conditionality of the PBF?**				
Always	-	-	56	75.7
Sometimes	-	-	18	24.3
**Do you take the opportunity to provide oral care for these children?**				
Yes	5	6.8	21	28.4
No	69	93.2	53	71.6
**If not, why?**				
Never thought about it	14	18.9	25	33.7
Lack of time	11	14.9	11	14.9
It is not the nurseʼs job	-	-	5	6.8
Lack of collaboration between programs	37	50.0	9	12.1
Other	7	9.5	3	4.1
**Do you evaluate the oral health conditions of…**				
Children covered by the PBF?	-	-	32	43.2
Children not covered by the PBF?	-	-	47	63.5
Do you refer for dental appointments…				
Children covered by the PBF?	-	-	47	63.5
Children not covered by the PBF?	-	-	56	75.7
**Do you advise parents/caregivers to seek oral care?**				
Yes	-	-	59	79.7
No	-	-	15	20.3

ESF = Estratégia Saúde da Família (Family Health Program)

PBF = Programa Bolsa Família

*Bolsa Família* Program supervisors were predominantly nurses (95.9%,n = 71) and female (89.2%;n = 66). The average age in this group was 41.9±9.2 years (range:28–65) (Table 1). Nearly all *Bolsa Família* Program supervisors (97.3%;n = 72) agreed with the inclusion of oral health care in the conditionality of the *Bolsa Família* Program (Table 2), but 82.4% (n = 61) stated they did not provide specific oral health promotion or preventive activities to children covered by the *Bolsa Família* Program, most often due to lack of time (24.3%;n = 18), but 63.7% (n = 47) reported referring children covered by the *Bolsa Família* Program under seven years old for dental care. However, children in the routine care setting were more often referred than children covered by the *Bolsa Família* Program (75.7%; n = 56;*p*≤0.001) (Table 3).

Parents/caregivers were aged 33.7±9.5 years on average (range:16–69) and approximately one third (30.2%;n = 116) were not married. Many (44%;n = 169) were elementary school dropouts. Almost all were women (97.9%;n = 376) and 85.1% (n = 327) identified themselves as the mother of the child covered by the *Bolsa Família* Program (Table 1). Most parents/caregivers (91.9%;n = 353) agreed with the health-related *Bolsa Família* Program conditionalities, especially because they considered check-ups important for the childʼs development (44.5%;n = 171). Practically all parents/caregivers (99.2%; n = 381) agreed that oral health care is important and 99.7% (n = 383) would like their children to be seen regularly by a dentist (Table 2).

Although promoting oral health activities (e.g., fluoride therapy programs, oral hygiene instruction, oral health education activities) targeting all children are some of the Family Oral Health team duties, very few dentists include such activities in their work routine. Only 43% of dentists interviewed reported that they developed school-based oral health promotion activities. It is important to note that most children covered by the *Bolsa Família* Program are not in school yet, do not attend pre-school, and therefore would be out of reach of these programs. In addition, only 25.7% of dentists reported developing these activities outside the school and kindergarten, (e.g., public health facilities) and an even lower proportion of dentists (6.8%) reported developing oral health promotion activities with children covered by the *Bolsa Família* Program (Table 3).

The inclusion of oral health care in the conditionality of the *Bolsa Família* Program was deemed desirable by 88.8% (n = 341) of the interviewed parents/caregivers (Table 2). Reportedly, many children (59%; n = 224) had never received fluoride therapy and the vast majority (95.8%; n = 368) had never participated in educational and preventive oral health sessions during visits to the Public Primary Health Care facility. According to parents/caregivers, over two thirds of children (77.3%; n = 297) had never been to the dentist at the public primary health care facility, most often due to difficulties in accessing dental care services (40.6%;n = 156).

Two thirds of the dentists (74.3%;n = 55) and *Bolsa Família* Program supervisors (75.7%;n = 56) performed no systematic efforts to provide oral health promotion or prevention to parents/caregivers of children covered by the *Bolsa Família* Program. Many of the dentists (40.5%;n = 30) mentioned lack of integration between the *Bolsa Família* Program and the Family Health Program as the main reason for not developing oral health promotion or prevention activities. Many of the *Bolsa Família* Program supervisors stated they had never thought about it (35.1%;n = 26) (Table 3).

When testing for correlations between the variables in the dentist group, a significant relationship was noted between the variables “oral health care check-ups for children covered by the *Bolsa Família* Program are important” and “oral health care should be made a *Bolsa Família* Program conditionality” (*p*≤0.001, Pearson’s chi-square test). The variables “agree with the existence of health-related conditionalities” and “agree with the inclusion of oral health care in the conditionality of the *Bolsa Família* Program” were significantly related (*p* = 0.002), but no relation was observed between the variables “believes that the infrastructure of the health care facility is appropriate for oral health services” and “agrees with the inclusion of oral health care in the conditionality of the *Bolsa Família* Program” (*p* = 0.361). When comparing dentists sorking exclusively for the Family Health Program to those working a third shift elsewhere (both with the same work shift at the Family Health Program), a significant difference (Pearson chi-square, p = 0.031) was found regarding “taking advantage of regular visits to the health unit by children covered by the *Bolsa Família* Program to provide them with oral health care (health promotion and prevention)”, where those who also work elsewhere, are more likely to perform such care than those who only work at the Family Health Program. When comparing orthodontists and/or cosmetic dentists with all other dentists working for Family Health Program, it was noted that the former were less likely to perform health promotion and preventive activities for children outside daycare (chi-square, p = 0.044), as well as seem to know less about BF conditionalities (chi-square, p = 0.05) than the latter.

## Discussion

This is the first study to evaluate the provision of oral health care by the Family Health Program to children under seven years old covered by the *Bolsa Família* Program. Studies have shown that the positive results of the *Bolsa Família* Program are to some extent associated with compliance to the Bolsa Família health conditionality, which is conducted by Family Health Program teams in the public primary health care facilities [13, 25]. In fact, according to Rasella [13], the quality and accessibility of primary health care services are vital to the success of Conditional Cash Transfer Programs in general. However, if the synergy between public services is weak, programs such as the *Bolsa Família* become considerably less effective [25]. Recent reviews of conditional cash transfer programs indicate an increase in the use of health services, with a positive impact on health outcomes [13]. However, no country with Conditional Cash Transfer Programs has, to date, included oral health care as a health conditionality [13]. As for Brazil, little is known about the provision of oral health care by the Family Health Program to children under seven years covered by the *Bolsa Família* Program. The present study is an attempt to fill this gap.

Children growing up in extreme poverty are generally at greater risk of developing chronic conditions such as stunting and dental caries and are more likely to suffer lifelong limitations imposed by such conditions [26]. The consequences of social inequality are particularly evident in the oral health of vulnerable populations such as children under seven years old covered by the *Bolsa Família* Program [27]. For example, the average DMF index (decayed, missing, filled teeth) of children aged 4–8 years born to and raised by families benefited by the *Bolsa Família* Program (4.77) is almost twice the average for Northeastern Brazil, which displays the country’s worst oral health indicators [7]. This is supported by Oliveira *et al*. [28] who found caries to be more prevalent and severe in school children covered by the PBF than in children not registered with the program. These authors also found that children covered by the *Bolsa Família* Program also had the lowest levels of attendance to dental services [28]. On the other hand, programs, such as the American Head Start and Early Head Start programs, which target low-income families and have a dental component (e.g., early dental screening), have shown to increase the usage of oral health care services [29, 30].

Our study found that the proportion of dentists including oral health promotion activities in their daily routine is very low. This proportion is even lower among dentists specifically targeting beneficiaries of the *Bolsa Família* Program. While working a third shift was associated with increased engagement in oral health promotion activities, having an orthodontics and/or cosmetic dentistry specialty was associated with a lower engagement in oral health promotion activities. These results point out to the need of implementing continuing education programs for dentists that are not engaging in oral health promotion activities. This initiative could help dentists promote oral health, and improve the dental care currently provided for the community. It should be noted that dentists working for the Family Health Program generally do not perceive children covered by the Bolsa Família Program as a specific risk group, as such children often receive treatment through school campaigns and other initiatives without being identified as *Bolsa Família* Program recipients. The same pattern was observed for *Bolsa Família* Program supervisors. The latter provided more health promotion and prevention care, and referred more children for dental treatment in the routine care setting than when the children were identified as beneficiaries of the *Bolsa Família* Program.

Our results showed that although most dentists agreed that children under seven years covered by the *Bolsa Família* Program should receive comprehensive oral health care, due to both their age (ideal for initiating prevention and establishing healthy habits) and socioeconomic vulnerability, these children were not prioritized in the family health dentists' work routine. This represents a missed opportunity to promote the oral health for this vulnerable group. The childrenʼs regular attendance of Public Primary Health Care facilities as part of the conditionality of the *Bolsa Família* Program may be used to increase access to oral health care. In addition, a change in priorities would make these children more accessible to Family Health Program professionals since many children under seven years are not enrolled in kindergarten or preschool and therefore do not benefit from Family Health Program-sponsored campaigns in public schools, when those are performed.

It is rather intriguing that, despite recognizing the urgent oral health care needs of children covered by the *Bolsa Família* Program, and being aware of the opportunity for referral provided by the children’s regular contact with Family Health Program professionals, dentists made little or no effort to promote or provide oral health care to this risk group. One of the reasons given by a significant number of dentists and *Bolsa Família* Program supervisors was that they saw no close connection between the Family Health Program and the *Bolsa Família* Program and many had never considered giving special attention to this risk group. This finding reveals serious flaws in the working and planning processes of the Family Health Program when viewed in light of the tenets of the National Primary Health Care Policy (PNAB) [12] and the National Oral Health Care Policy (PNSB) [31] set forth by the Ministry of Health.

Brazilian primary care is supported by the pillars of universality, accessibility, bonding, continuity, integration, accountability, humanization, equity and social participation. Brazil’s National Primary Health Care Policy fosters individual and collective health actions with the purpose of developing integrated care capable of positively impacting citizens’ health status, autonomy and social determinants of health towards greater equity [12].

In accordance with the Brazilian’s National Oral Health Care and National Primary Health Care Policy’s guidelines, oral health teams should i) plan and provide effective oral health care (health promotion and protection, disease prevention, diagnosis, treatment, follow-up, rehabilitation and maintenance of health) for all individuals, families and risk groups, ii) coordinate and participate in campaigns and projects of oral health promotion and prevention, and iii) collaborate with other Family Health Program professionals in the building of a multidisciplinary framework of integrated health care [12,32]. Early childhood is an important time for oral health promotion. The earlier the enrollment in preventive programs, the better the protection against the future development of dental caries even in children with unfavorable socioeconomic conditions [32]. This should be one of the aims of the Family Health Program Oral Health Teams.

The guidelines expounded above point to a Public Primary Health Care system centered on population health care needs. However, according to Ramos and Cuervo [33], at the Public Primary Health Care facilities attended by beneficiaries of the *Bolsa Família* Program, this outlook is obscured by an overemphasis on bureaucratic aspects and a lack of awareness of the essential social and political role of the *Bolsa Família* Program. The authors added that, on part of the Family Health Program, health-related *Bolsa Família* Program conditionalities translate into little more than the collection of statistical indicators, such as weight, height, vaccination status and breastfeeding indices. Many Family Health Program professionals do not think of the *Bolsa Família* Program as a potential key instrument for the promotion of health, let alone oral health. As for the latter, professional motivation and government pressure have been insufficient to explore the potential for this type of intervention and municipal agencies lack clear guidelines for the supervision of oral health promotion, prevention and treatment of children under seven covered by the *Bolsa Família* Program. Although the guidelines of the Bolsa Familia Program [34] recommend that families covered by the Program attend health education sessions on breastfeeding, pediatric nutrition and care, none of the study participants' reported attending these activities.

According to Adato *et al*. [21], though the mechanism of the cash incentive is a powerful one, and program impact studies show that many people do respond as expected, ongoing gaps in the use of health services have not received adequate attention. By focusing primarily on successes, implementers miss the opportunity to make programs more effective and more appropriate for their constituencies, and researchers miss the opportunity to better explain their findings.

Most of the interviewees expressed the belief that children covered by the *Bolsa Família* Program are unlikely to receive regular oral health check-ups unless oral care is made a *Bolsa Família* Program conditionality and thus agreed with this proposal. However, the interviewees did not seem to be aware that the provision of oral health care to vulnerable groups (e.g., children covered by the *Bolsa Família* Program) has always been a mandate of the Family Health Program. This raises the question of whether the inclusion of oral care in the conditionality of the PBF will bring about the desired change, and whether dental care is the only form of health service neglected by Family Health Program teams. The authors believe that, while desirable, the inclusion of dental care in the conditionality of the *Bolsa Família* Program may not solve the problem, but might instead strengthen the tendency to rely solely on rules and regulations to change behaviors and realities. Better results would likely be achieved by focusing efforts on the improvement of Public Primary Health Care infrastructure, continuing hands-on professional training, humanization and the ability to identify and satisfy population health care needs.

Considering the formal commission of the Family Health Program to provide primary health care—including oral care—to the Brazilian population as a whole and the emphasis of the National Oral Health Care and National Primary Health Care Policys guidelines on population-based care, failure to identify children under seven years of age covered by the *Bolsa Família* Program as a risk group, thus a priority, goes against their job description. Our findings not only confirm the relevance of including pediatric oral health care in the conditionality of the *Bolsa Família* Program, making the health care system more equitable, but, more importantly, allow to infer that significant changes in currently counterproductive processes and behaviors are unlikely to happen without continuing professional training of Family Health Program professionals in addition to an acute awareness of local population health care needs.

Conditionality in Conditional Cash Transfer Programs has the aim of helping the assisted population to develop human/social capital [21]. However, Conditional Cash Transfer Programs have been criticized because provision of incentives for individuals to change behavior might not work without supply side investments [35]. According to Fernald [25] Conditional Cash Transfer Program in countries without nationalized health care might not be as effective. In that line of thinking, it might be inferred that in countries where the national health care system is not working at its full potential, the effectiveness of its Conditional Cash Transfer Program and the generation of social/human capital might not be fulfilled.

In this study, no collaboration was observed between the Family Health Program (*Estratégia Saúde da Família*) and the *Bolsa Família* Program (*Programa Bolsa Família*) with regard to the provision of oral health care for children under seven at public primary health care facilities in Fortaleza. No systematic oral health promotion and prevention initiatives were specifically directed at children covered by the *Bolsa Família* Program or their parents/caregivers.

However, considering the protective effect of preventive and restorative oral health care in children, as well as the well-documented potentiating effect of intersectorality on health care outcomes in general, a closer interaction between the *Bolsa Família* Program and the Family Health Program would help reduce inequalities in current oral health care for children under seven years of age. The following actions are suggested: i) inclusion of oral health care for children under seven years of age as a *Bolsa Família* Program conditionality; ii) improvements in Family Health Program organization and infrastructure (including human resources and supplies) to reflect National Primary Health Care Policy guidelines; iii) continued training of Family Health Program professionals to capacitate them towards the needs of children covered by the *Bolsa Família* Program, as well as ways to improve children oral health; iv) creation of coordination programs, at all public administrative levels (Municipal, State and Federal), to enhance the interaction between *Bolsa Família* and Family Health Programs towards actions aimed at *Bolsa Família* Program families—allowing optimization of resources and better health outcomes for the population; and v) enforcement of working and planning processes in accordance with the National Primary Health Care Policy (PNAB) and the National Oral Health Care Policy (PNSB) guidelines.
